# Modulating p56Lck in T-Cells by a Chimeric Peptide Comprising Two Functionally Different Motifs of Tip from *Herpesvirus saimiri*

**DOI:** 10.1155/2015/395371

**Published:** 2015-10-11

**Authors:** Jean-Paul Vernot, Ana María Perdomo-Arciniegas, Luis Alberto Pérez-Quintero, Diego Fernando Martínez

**Affiliations:** ^1^Fisiología Celular y Molecular, Facultad de Medicina, Universidad Nacional de Colombia, Bogotá 11001, Colombia; ^2^Instituto de Investigaciones Biomédicas, Facultad de Medicina, Universidad Nacional de Colombia, Bogotá 11001, Colombia

## Abstract

The Lck interacting protein Tip of *Herpesvirus saimiri* is responsible for T-cell transformation both* in vitro *and* in vivo*. Here we designed the chimeric peptide hTip-CSKH, comprising the Lck specific interacting motif CSKH of Tip and its hydrophobic transmembrane sequence (hTip), the latter as a vector targeting lipid rafts. We found that hTip-CSKH can induce a fivefold increase in proliferation of human and* Aotus* sp. T-cells. Costimulation with PMA did not enhance this proliferation rate, suggesting that hTip-CSKH is sufficient and independent of further PKC stimulation. We also found that human Lck phosphorylation was increased earlier after stimulation when T-cells were incubated previously with hTip-CSKH, supporting a strong signalling and proliferative effect of the chimeric peptide. Additionally, Lck downstream signalling was evident with hTip-CSKH but not with control peptides. Importantly, hTip-CSKH could be identified in heavy lipid rafts membrane fractions, a compartment where important T-cell signalling molecules (LAT, Ras, and Lck) are present during T-cell activation. Interestingly, hTip-CSKH was inhibitory to Jurkat cells, in total agreement with the different signalling pathways and activation requirements of this leukemic cell line. These results provide the basis for the development of new compounds capable of modulating therapeutic targets present in lipid rafts.

## 1. Introduction

Selective phosphorylation of tyrosine residues in the T-cell receptor- (TCR-) associated CD3 complex and *ζ* chains follows TCR engagement and activation by the MHC-peptide complex [1]. Tyrosine phosphorylation is mediated by protein members of the nonreceptor Src tyrosine kinase family, mainly p56Lck (Lck) and p59Fyn (Fyn) [2]. Lck is lymphoid-specific and essential for T-cell development and function [3, 4]; it associates with surface molecules such as CD2, CD4, CD8, CD45, and IL-2 receptor [4–6]. Lck can be either phosphorylated by serine-threonine or tyrosine kinases but it is well known that its activity is mainly positively and negatively regulated by tyrosine phosphorylation in positions 394 and 505, respectively [7, 8]. Stimulation of T-cell lines defective in Lck expression has shown an abnormal tyrosine phosphorylation pattern of downstream protein targets [9]. Lck has acquired importance as a therapeutic target for regulating T-cell response due to this central role in T-cell function [10–12].

Lck, as well as other Src family members, is also the target of viral proteins as a strategy for lymphotropic viruses [13], such as human immunodeficiency virus [14], Epstein-Barr virus [15], or* Herpesvirus saimiri* (HVS), to escape immune control and maintain latency [16]. In particular, HVS is a lymphotropic *γ*-herpesvirus, which is nonpathogenic in its natural host* Saimiri sciureus*, but in some New World primate species induces fulminant T-cell lymphomas [17]. HVS infection has been used* in vitro *in human and non-human primates (*Aotus* spp.) as a strategy for T-cell transformation [18–20]. This would suggest that transformation mechanisms in human and* Aotus *T-cells could have the same molecular basis.

Two HVS gene products, Tip (tyrosine kinase interacting protein) and StpC (*Saimiri *transforming protein), seem to be essential for the observed oncogenic phenotype [21]. Tip was able to induce T-cell lymphoma in transgenic mice and is therefore very likely to be responsible for the oncogenicity in T-cells [22]. A mechanistic model in which Tip participates in TCR signalling and CD4 downregulation has been proposed to explain HVS-infected T-cell longevity with consequences on viral persistence and pathogenesis [23]. Some studies have shown that Tip specifically associates with Lck, increasing its phosphorylation and activity and thus T-cell proliferation [24–26]. Moreover, Tip has been extensively implicated in oncogenic transformation [13, 27].

Nevertheless, in Jurkat T-cell lines or primary T-cells immortalized by lentiviral transduction, other authors have shown that Tip induces downregulation of Lck and an overall decrease in cellular tyrosine phosphorylation of several proteins, including Lck and ZAP70 [16, 28]. Early work has shown differences in signalling molecule expression and altered requirements for Jurkat cells activation [29], explaining in part the opposite effect of Tip in primary T-cells versus Jurkat cells or immortalized T-cells.

The Lck-binding domains (LBD) of Tip have been mapped to the carboxyl terminal portion of the molecule involving two independent binding motifs, SH3B (SH3 binding) and CSKH (C-terminal Src kinase homology). They are included within a highly conserved region between amino acids 146 to 182 [30–33]. The SH3B motif (residues 172 to 182) binds to the SH3 domain on Lck, while the CSKH motif (residues 146 to 155) binds to the kinase catalytic domain [24]. Interestingly, mutants of Tip, containing either only SH3B or only CSKH domains, bind to Lck although to a reduced extent [32]. Also a truncated form of Tip lacking SH3B is still able to induce lymphomas* in vivo* [34] and coexpression of Lck with a Tip mutant lacking SH3B stimulated tyrosine phosphorylation of cellular proteins [32]. This suggests a suitable and sufficient role of the CSKH domain for Lck binding, T-cell signalling, proliferation, and eventually cell transformation.

As it would be expected for a protein regulating Lck, Tip has been shown to be constitutively present in lipid rafts [23, 35], a signalling platform for T-cell activation [36, 37], where early signals are induced allowing subsequently specific gene expression and proliferation [26, 38, 39]. The carboxyl terminal hydrophobic (hTip) sequence of Tip is responsible for its localization to lipid rafts [40]. In the present work, we have explored the intriguing possibility of inducing T-cell activation and proliferation by using a short Lck binding motif of Tip (CSKH) properly delivered to lipid rafts. In fact, the hTip-CSKH chimera was delivered to detergent resistant membranes allowing us to specifically target Lck, to induce intracellular signalling and T-cell proliferation. Of great relevance, this work suggests that using a lipid raft targeting sequence from the transforming Tip protein could be a novel strategy to activate or inhibit signal transduction pathways, in other cell types and conditions, in which lipid raft dynamic is involved.

## 2. Methods

### 2.1. Peptide Synthesis and Characterization

Peptides were synthesized by solid phase as previously described [41]. The hTip sequence corresponds to the carboxyl-terminal residues 232–250 (CLVVVILAVLLLVTVLSIL); the CSKH motif EDLQSFLEKY plus a 6-residue extension (PPDFRK) adjacent to the CSKH motif was used as the hTip cargo, forming the chimera (CLVVVILAVLLLVTVLSILEDLQSFLEKYPPDFRK), here called hTip-CSKH. hTip, CSKH, and a chimeric peptide with the CSKH sequence in a scrambled configuration (hTip-CSKHsc) were also used in some experiments as controls. Peptides were analyzed by RP-HPLC and mass spectrometry to ascertain molecular weight and purity.

### 2.2. Blood Samples, Cell Isolation, and Cell Line

Human blood samples were obtained from healthy donors after informed consent.* Aotus nancymaae *from the Colombian Amazon region were housed according to NIH guidelines for animal handling. Both human and* Aotus *peripheral blood mononuclear cells (PBMC) were isolated by Ficoll-Hypaque density gradient centrifugation (Sigma-Aldrich Co., St. Louis, MO, USA). The Jurkat T-cell leukemia cell line (ATCC) was cultured under standard conditions. Cell viability was routinely assessed by Trypan-blue dye exclusion and only samples having >90% viability were further used.

### 2.3. *Aotus nancymaae *Lck Sequence Determination

RNA from 1 × 10^7^ lymph node cells was extracted by TRIZOL (Gibco, Invitrogen Corp., New York, NY, USA) as described elsewhere. Lck coding and proximal 5′ and 3′ UTR regions were amplified by RT/PCR using SuperScript III One Step RT/PCR with Platinum Taq (end point) (Invitrogen Life Technologies, Carlsbad, CA, USA) with sense primer LckRs17 (5′GCCTGGACCATGTGAAT3′) and antisense primer LckRa20 (5′TGACTATGGCACAAGAACTC3′). Three clones were obtained and sequenced using 6 different primers: LckRs17, LckRa20, M13 forward, M13 reverse primers, and two internal primers LckIntF (5′TGGACAGTTCGGGGAGG3′) and LckIntR02 (5′ATGATGTAGATGGGCTCCT3′). Sequencing was performed with BigDye 3.1 sequence kit and analyzed in ABI PRISM 310 genetic analyzer, with the ABI PRISM Sequencing Analysis 3.3 MT Navigator 1.0.2. (Perkin Elmer, Foster City, CA, USA) and Chromas 1.45 software. Other sequencing was done by MACROGEN Inc. Overlapping sequences were aligned using ClustalX 1.83 software and analyzed by GeneDoc software (multiple sequence alignment editor, version 2.5.000). The sequence submitted to GenBank (accession number:* AY821852*) was the consensus sequence from the three clones. Human,* Saimiri sciureus, *and* Aotus nancymaae *(accession numbers:* NP_005347*,* CAC38871*, and* AAV70114*, resp.) protein sequences were compared by using the same software. Sequences used were found in the GenBank protein databases.

### 2.4. Peptide FITC-Labeling and Fluorescence Microscopy

hTip-CSKH and scrambled CSKH peptides were labeled with fluorescein isothiocyanate (FITC). Shortly, 4.75 mL of a 0.0125 mM solution of peptide was incubated 24 h in 0.1 M carbonate-bicarbonate buffer with 250 *μ*L of a 2.5 mM FITC solution in DMSO. Dialysis was performed during 48 h against 0.1 M ammonium chloride in 1% DMSO (membrane of exclusion limit of 3.5 kDa; Spectrum Laboratories Inc., Rancho Dominguez, CA, USA). 10^5^ human PBMC resuspended in RPMI 1640 supplemented with 10% FCS, 10 mM HEPES, and 1 mM sodium pyruvate (complete medium) for 1 h at 37°C were incubated with 40 *μ*M of FITC-labeled hTip-CSKH or CSKH(Scr). Cells were washed in PBS and resuspended in 20 *μ*L of media (150 mM NaCl, 50% glycerol) and mounted in a glass microscope slide with a coverslip and observed in a Nikon C-1plus fluorescence microscope. Digital photographs were taken with a Sony DSC-P73 digital camera.

### 2.5. Lymphocyte Proliferation Assays

A total of 5 × 10^4^ PBMC/well were cultured for 1 h at 37°C and 5% CO_2_ in complete medium in the absence or presence of different peptide concentrations (as indicated in each figure) in 96-well flat-bottomed microplates (Linbro, Aliso Viejo, CA, USA). In some experiments, PBMC were also stimulated with 2 *μ*g/mL PHA-P or 25 ng/mL PMA for 48 h or 72 h with or without addition of peptides. Afterwards, 0.5 *μ*Ci/well [methyl-3H]-thymidine (ICN Biomedicals, Inc., Irvine, CA, USA) was added to cultures for the last 18 h. Cells were then harvested (PHD Harvester, Cambridge Tech) in glass fiber strips (Cambridge Technology, Watertown, MA, USA) and assayed for [methyl-3H]-thymidine incorporation by liquid scintillation counting *β*-scintillation system Beckman LS 6500 (Beckman Instruments, Fullertown, CA, USA).

Peptides-treated or peptides-untreated Jurkat cells (5 × 10^4^) were stimulated with 2 *μ*g/mL ionomycin or 2 *μ*g/mL PHA-P and processed as described above for primary cells. The stimulation index percentage (SIP) was defined as being the ratio of mean [methyl-3H]-thymidine (counts per minute) incorporated in the presence of a peptide to that incorporated in the absence of peptide (×100).

### 2.6. Protein Tyrosine Kinase Immunostaining

PBMC from humans were preincubated (or not) for 1 h with 60 *μ*M hTip-CSKH and further stimulated with PHA-P (5 *μ*g/mL) for 15, 30 or 60 min. Stimulation was stopped by ice-cooling the cultures; these cells were then washed twice in ice-cold PBS and cell extracts were prepared by a 20-minute incubation in lysis buffer (20 mM Tris-Cl pH 8.0, 276 mM NaCl, 10% glycerol, 1% NP40, 1 mM PMSF, 10 *μ*g/mL aprotinin, 10 *μ*g/mL leupeptin, 1 mM Na3VO4, 10 mM NaF, and 2 mM EDTA). Extracts were centrifuged for 15 min at 10,000 g and 4°C; supernatants were recovered and subjected to 10% SDS-PAGE as described elsewhere. Proteins were then transferred to PVDF membranes (Immobilon-P, Millipore Corp., Bedford, MA, USA). Immunodetection was performed with anti-Lck (clone MOL171) antiphosphotyrosine (Clone PY20) (Pharmingen, San Diego, CA, USA). In other sets of experiments, human PBMC were stimulated with PHA-P or hTip-CSKH and cell extracts prepared as described above. Proteins were then transferred to PVDF membranes and immunodetection was performed with anti-Fyn (AHO0482, Invitrogen) and anti-*β*-Actin (sc-47778, Santa Cruz Biotechnology). For data analysis, p56Fyn and p59Fyn bands were normalized to *β*-actin.

### 2.7. Downstream Signaling after hTip-CSKH Stimulation

For ERK 1/2 phosphorylation, human PBMC were stimulated for 2 h with the different peptides or with PHA-PMA as described above. Cells extracts were prepared as above and assayed by WB with anti-p-ERK1/2 (sc-7383, Santa Cruz Biotechnology) and anti-ERK1/2 (sc-7383, Santa Cruz Biotechnology). Erk phosphorylation was normalized to total ERK1/2.

### 2.8. Biochemical Isolation of Detergent Resistant Membranes (DRMs)

6.5 × 10^7^ human PBMC were treated with 40 *μ*M of hTip-CSKH in serum-free RMPI-1640 during 1 hour at 37°C. The cells were washed in medium and the membranes were extracted during 20 min in 200 *μ*L of ice-cold lysis buffer containing Tris 25 mM pH 7.4, 150 mM NaCl, 2 mM EDTA, 0.5% Triton-X100, 1 ug/mL leupeptin, 1 ug/mL pepstatin, 4 ug/mL aprotinin, 1 mM PMSF, 1 mM Na3VO4, and 50 mM NaF. For sucrose density gradients, cell extracts were mixed with 250 *μ*L of 80% sucrose in TNE buffer (Tris 25 mM pH 7.4, 150 mM NaCl, and 2 mM EDTA) and overlayed with 730 *μ*L of 35% sucrose and 320 *μ*L 5% sucrose and centrifuged at 200,000 g during 5 h, 4°C (Beckman Optima, rotor TLS-55). Eight fractions of equal volume were recovered from the top of the gradient, diluted with 1 mL of TNE, and detergent resistant membranes in each fraction were precipitated by ultracentrifugation at 100,000 g during 45 min, 4°C. The pellets were extracted in 20 *μ*L of Laemmli buffer: half used for WB and Flotillin-2 detection and the other half for SDS-PAGE in Tris-Tricine buffer for peptide identification as described [42]. To facilitate the identification of the peptides in this system, erythrocyte membranes were prepared in parallel and incubated with the different peptides used. Peptides were identified by sample separation in Tris-Tricine SDS-PAGE.

### 2.9. PBMC Surface Marker Characterization after hTip-CSKH Treatment

PBMC were incubated for 24 h in the absence or presence of 60 *μ*M hTip-CSKH peptide, PBS washed, suspended in 0.5% BSA-PBS solution, and further incubated for 20 min at RT with the respective monoclonal antibody. Anti-CD3 PE-conjugated antibody (clone UCTH1) was purchased from Sigma (Sigma-Aldrich Co., St. Louis, MO, USA); and anti-TCR *α*/*β* FITC-conjugated antibodies were purchased from Pharmingen (San Diego, CA, USA). All incubations with fluorochrome-tagged antibodies were done in the dark. Then, cells were washed with 2 mL of PBS and centrifuged. After a final wash, cells were suspended in 0.5 mL PBS and immediately read in a FACScan flow cytometer (Beckton Dickinson, BD Biosciences, San Jose, CA, USA).

### 2.10. Statistical Analysis

Paired statistical analyses were performed using Student two-tailed *t*-test. Paired two-tailed *t*-test analyses were performed for the data comparing values obtained from nontreated and hTip-CSKH or hTip-treated cells at the same time points.

## 3. Results

### 3.1. High Identity in Lck and Similarity in T-Cell Proliferation in Response to hTip-CSKH between* Aotus *and Humans

The* Aotus* primate experimental model has proved to be useful for basic immunology and vaccination studies. The establishment of similarities is essential for physiological studies involving immune modulation, although some differences in T-cell response have been found [43]. Previously, we found that* Aotus *Lck was more related to* Saimiri sciureus *than to human Lck. Nevertheless, high identity (>98%) was also observed between human and* Aotus *Lck protein sequences (Supplementary Figure 1, see Supplementary Material available online at http://dx.doi.org/10.1155/2015/395371).  Functionally important sequences for molecular docking (i.e., CENCH motif for CD4/CD8 coreceptors binding and SH2 phosphotyrosine binding domain) or catalysis (ATP binding region, 364D and 273K residues) were highly conserved. Differences between human and* Aotus *Lck sequences rely only on 12 residues.

**Figure 1:**
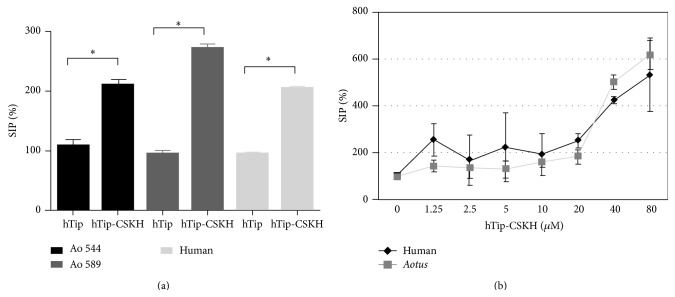
hTip-CSKH induced cell proliferation in human and* Aotus* cells. (a) PBMC from two* Aotus nancymaae *and one human donor were treated with 40 *μ*M hTip or hTip-CSKH peptides and assayed for [3H]-thymidine incorporation after 48-hour treatment. (b) Human and* Aotus *PBMC exposed to several hTip-CSKH concentrations were assessed for [3H]-thymidine incorporation after 72-hour treatment. Stimulation index percentage (SIP) is shown (see Section 2). Student's *t*-test statistically significant values (*p* < 0.05) are marked with asterisk (*∗*).

Cell proliferation in* Aotus* and humans has different requirements: while* Aotus* PBMC respond poorly to PHA-P, they readily respond to favin stimulation [43]. On the other hand, it is well known that human lymphocytes require at least two different signals (signals 1 and 2) for proliferation [44]. These signals can be simulated* in vitro* by the combination of calcium influx (signal 1) and PKC activation (signal 2) or PHA-P stimulation (both signals). Interestingly, incubation of* Aotus* or human PBMC with hTip-CSKH (40 *μ*M) for 48 h induced increased proliferation (2-3 times) (Figure 1(a)). The increased proliferation was dependent on the cargo sequence since proliferation of PBMC treated with hTip alone or the chimeric CSKH scrambled peptide (hTip-CSKHsc) was as low as that of control cells without peptide (not shown). Some variability in* Aotus* response to hTip-CSKH compared to humans was observed, but this is probably due to what we have previously described for this experimental model [43]. The hTip-CSKH effect is then similar to PHA-P, that is, inducing proliferation by its own, although to a lesser extent. The result observed with hTip-CSKH was stronger and dose-dependent in both human and* Aotus *PBMC when proliferation was assayed after 72-hour incubation and with different peptides concentrations (Figure 1(b)). Increased proliferation was initially observed between 20 and 40 *μ*M but, remarkably, a sixfold proliferation induction was observed at 80 *μ*M hTip-CSKH and 72-hour incubation in both human and* Aotus* T-cells without losing cell viability.

### 3.2. hTip-CSKH Induces Proliferation in Human T-Cells Independently of Signal 2

**Figure 2:**
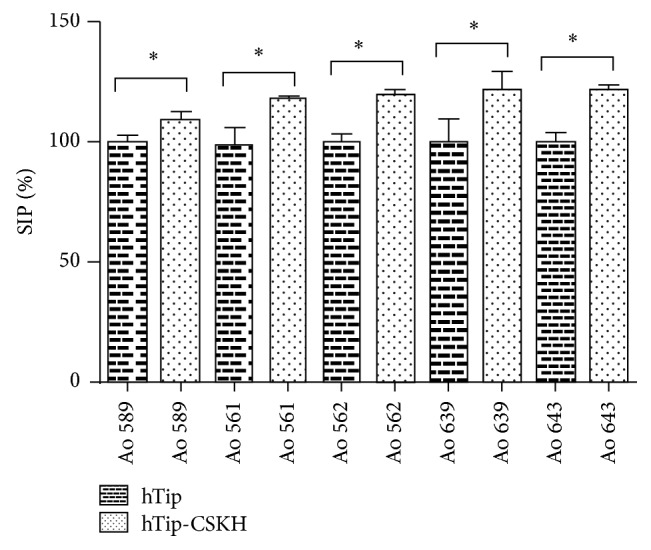
hTip-CSKH even induced a slight increase in proliferation in PHA-P-stimulated* Aotus nancymaae* PBMC. Five* Aotus nancymaae *PBMC samples were treated with hTip and hTip-CSKH peptides (40 *μ*M); after 1-hour incubation, 2 *μ*g/mL PHA-P was added to the culture. [3H]-thymidine incorporation was assessed after 72 h of culture. Student's *t*-test statistically significant values (*p* < 0.05) are marked with asterisk (*∗*).

As indicated, proliferation induced by the polyclonal T-cell activator PHA-P in* Aotus* PBMC is lower compared to humans. Also human PBMC proliferation induced by hTip-CSKH was lower (40% or more dependent on the conditions) than that usually induced by PHA-P, suggesting different mechanisms of induction and/or different strength of activation. To address this issue initially, we examined hTip-CSKH proliferative effect on previously PHA-P-stimulated* Aotus *T-cells. In spite of the variability reported previously and observed above, there was a statistically significant increase in proliferation induced by hTip-CSKH (Figure 2) in 5* Aotus* PBMC samples that have been previously activated by PHA-P. As expected, this increase was low (11–22%) but statistically significant. Control hTip peptide treatment did not increase proliferation, confirming that the proliferative effect is therefore cargo specific. Our results show that T-cells that have been previously stimulated by the strong activator PHA-P can still be further activated by the chimeric peptide hTip-CSKH, suggesting either that stimulation with PHA-P is not maximal in this condition and hTip-CSKH delivers a synergic proliferation signal or that hTip-CSKH signals through other pathway(s).

**Figure 3:**
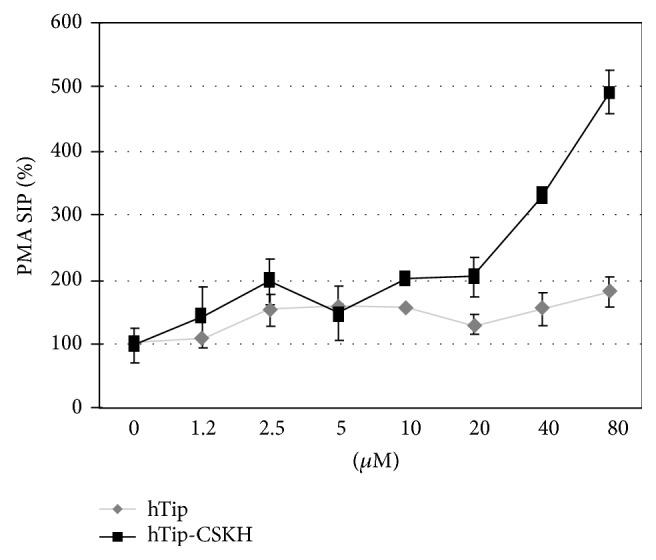
hTip-CSKH induced similar 5-fold increased proliferation in PMA-stimulated PBMC. Human PBMC exposed to different hTip-CSKH or hTip concentrations for 1 h were stimulated with 25 ng/mL PMA. Cells were assessed for [3H]-thymidine incorporation after 72 h. Stimulation index percentage (SIP) is shown.

To further explore this topic, we used the classical protein kinase C (PKC) activator phorbol-12-myristate-13-acetate (PMA) which induces signal 2 [44], bypassing cell surface receptors stimulation. We hypothesized that PKC activation will act as a complementary signal to hTip-CSKH. As expected, a relatively low cell proliferation was obtained when human PBMC were treated only with PMA. Addition of hTip-CSKH induced a dose-dependent increase in proliferation (starting at 20–40 *μ*M) with a maximum fivefold increase at 80 *μ*M when compared to PMA-treated cells; the control peptide hTip did not produce the same effect (Figure 3). The onset of cell proliferation and maximal response to hTip-CSKH was very similar to that obtained without PMA (compare Figures 1(b) and 3). Therefore, hTip-CSKH seems to be able to efficiently replace signal 2 on its own.* Aotus* PBMC were not used in these PMA experiments, since we have previously shown that under certain circumstances (and for unknown reasons) these cells can respond to either signal 1 or signal 2 [43].

### 3.3. hTip-CSKH Effect on the T-Cell Line Jurkat

**Figure 4:**
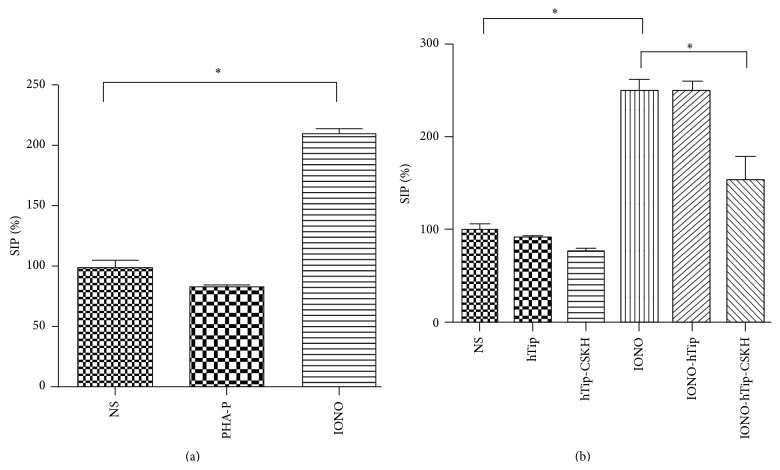
hTip-CSKH induced a decreased cell proliferation in Jurkat cells. (a) Jurkat cells were treated with PHA-P or ionomycin and assayed for [3H]-thymidine incorporation after 48-hour treatment. (b) Jurkat cells were treated with hTip, hTip-CSKH for 1 h, and then ionomycin-stimulated and assayed for [3H]-thymidine incorporation. Jurkat cells treated with each peptide alone or nontreated peptides were added as controls. Stimulation index percentage (SIP) is shown (see Section 2). NS: nonstimulated; Iono: ionomycin. Student's *t*-test statistically significant values (*p* < 0.05) are marked with asterisk (*∗*).

Jurkat T-cells were very interesting to further test hTip-CSKH effect, since TCR activation in this cell line causes growth inhibition, suggesting that these cells have different activation requirements [45]. Jurkat cells do not respond to PHA-P stimulation but respond adequately to calcium influx.  Figure 4(a) shows that Jurkat cells had a proliferation rate about two- to threefold higher in the presence of ionomycin than unstimulated cells. As it has been previously reported, PHA-P-stimulated cells showed a slight reduction in the proliferation rate (Figure 4(a)). Interestingly, hTip-CSKH treatment of Jurkat cells also showed a slight diminution in proliferation, similar to what we have seen before with PHA-P (compare Figures 4(a) and 4(b), 3rd column with NS controls). Control hTip peptide had no effect (Figure 4(b), 2nd column). Ionomycin-stimulated Jurkat cell treated with hTip-CSKH showed a strong reduction in proliferation rate while control hTip-treated cells showed no effect (Figure 4(b)). Together, these results have shown that if T-cells are able to proliferate in response to PHA-P (signals 1 and 2 induction), as in normal human and* Aotus* T-cells, then h-Tip-CSKH will also induce proliferation. In contrast, if stimulation through signal 1 inhibits proliferation (as in Jurkat cells), then hTip-CSKH will also be inhibitory. This would give support to our previous suggestion that signaling by hTip-CSKH peptide is strongly enough (and probably includes both signals) to induce proliferation on its own.

### 3.4. hTip-CSKH Can Be Identified in Detergent Resistant Membranes (DRMs)

**Figure 5:**
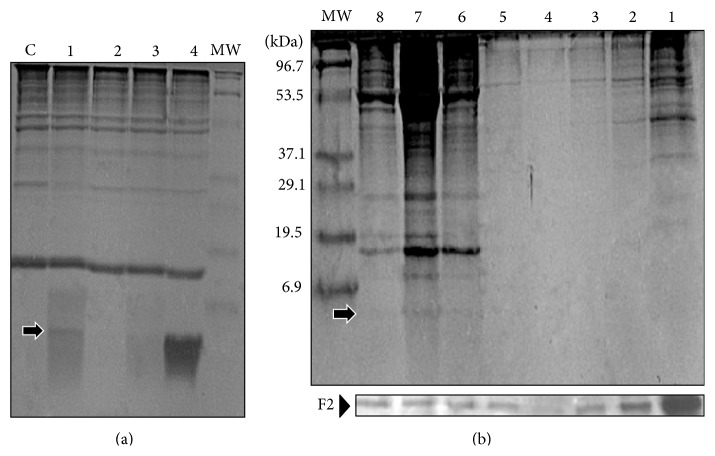
Tris-Tricine electrophoretic identification of hTip-CSKH or control peptides. (a) 10 *μ*g of the indicated peptides (1 = hTip-CSKH; 2 = hTip; 3 = hTip-CSKH-Scr; 4 = CSKH) was mixed with 10 *μ*L of a protein extract of red blood cells membranes. The gel was stained with Coomassie Brilliant Blue. The size of each molecular weight marker (MWM) is indicated in the right and the control (c) membranes without peptides in the left. The arrow indicates the band corresponding to hTip-CSKH molecular weight. (b) The membrane extract from hTip-CSKH-treated human PBMC was resolved by ultracentrifugation in a sucrose density gradient. Eight fractions were recovered as indicated in each line. Half of each fraction was used for Tris-Tricine electrophoresis for hTip-CSKH identification (the arrow marks the band corresponding to hTip-CSKH molecular weight). The remaining amount of the fraction was used for WB blot in order to know its Flotillin 2 content (bottom) and to differentiate between light (lanes 1–3) and heavy (lanes 5–8) fractions. The size of each molecular weight marker (MWM) is indicated in the left.

It has been previously reported that the transmembrane domain (amino acid residues 229–250) of Tip is required for its association with lipid rafts [35]. We then explored biochemically hTip-CSKH localization at the plasma membrane, specifically its association with DRM. Since DRMs localize into the low-density fractions in sucrose gradients, they have usually been associated with lipid rafts [37]. First, we tested an electrophoresis system (SDS-PAGE in Tris-Tricine buffer) having high-resolution power for peptides identification compared to classical SDS-PAGE. 10 *μ*g of each of hTip-CSKH, hTip, hTip-CSKHsc, or CSKH peptides was mixed with 10 *μ*L of an erythrocyte cell membranes extract. As it can be seen in Figure 5(a), hTip-CSKH, hTip-CSKHsc, and CSKH could be identified as diffuse and fast migrating bands below the 6,9 kDa MW standard (arrow). hTip could not be resolved probably because of its high hydrophobicity. We next prepare a Triton X-100 extract of hTip-CSKH-treated PBMC and fractionated it by sucrose density gradient centrifugation. Eight fractions were collected and assayed for hTip-CSKH (by Tris-Tricine SDS-PAGE) and Flotillin-2 (by SDS-PAGE and WB) presence. Flotillin-2, a marker of lipid rafts, was distributed mainly in fractions 1–3 and 6–8 (Figure 5(b), lower panel). A band corresponding to the hTip-CSKH MW was detected in fractions 6 and 7 (Figure 5(b), upper panel, arrow), showing hTip-CSKH localization in membrane rafts. The existence of a novel type of membrane raft-like microdomains (heavy DRM), containing a number of membrane signaling molecules, including LAT and Lck, was recently demonstrated [46]. The above evidence suggests that almost all of the chimeric hTip-CSKH is present in these novel heavy rafts, where it could interact with the Lck protein. Additionally, incubation of human PBMC with the FITC-labeled hTip-CSKH during 1 h and analysis by fluorescence microscopy showed a homogeneous surface peripheral localization and no intracytoplasmatic accumulation (Supplementary Figure 2). This shows that hTip-CSKH interacts with the cytoplasmic membrane and is localized in lipid rafts.

### 3.5. hTip-CSKH Targets Lck and Induces Its Early Activation

**Figure 6:**
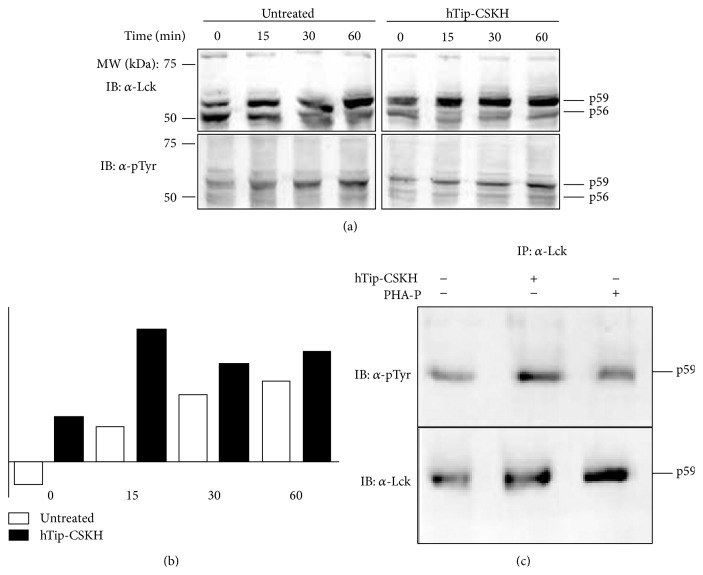
Time-course assay for Lck in human PBMC after cell stimulation. (a) PBMC either untreated (lanes 1–4) or treated (lanes 5–8) for 1 h with hTip-CSKH were stimulated with PHA-P for 0, 15, 30, and 60 min and then were lysed. Extracts were subjected to SDS-PAGE and proteins transferred to PVDF membranes. WB was performed with Lck (upper panels) and P-Y (lower panels) monoclonal antibodies. Two bands were clearly defined which correspond to the low molecular weight (p56) and the high molecular weight band (p59), respectively. (b) Quantification of p56 and p59 bands showed an increase of the p59/p56 ratio. White bars represent nontreated cells and black bars hTip-CSKH-treated cells p59/p56 ratio at different time points. (c) Lck immunoprecipitation of PBMC extracts after stimulation with PHA-P or hTip-CSKH for 30 min. WB was performed with monoclonal antibodies against pTyr and Lck as indicated.

The heavy lipid rafts are part of a TCR signaling platform involved in the early coordination of T-cell signaling events, namely, the activation of nonreceptor protein tyrosine kinases [46]. We have analyzed the hTip-CSKH effect on the lymphocyte-specific protein tyrosine kinase Lck, key molecule during T-cell activation, and hypothetical target of the hTip-CSKH chimeric peptide used here. Antibodies directed against human Lck and phosphotyrosine residues were used for assessing human Lck phosphorylation during time-course experiments, following T-cell short stimulation with PHA-P in the presence or absence of hTip-CSKH. Cell extracts were prepared after 0, 15, 30, and 60 min of stimulation; WB detection was simultaneously performed for Lck and phosphotyrosine residues. Defined bands of about 56 kDa and 59 kDa were evident in both blots (Figure 6(a)). When analyzing Lck staining at time point 0 (Figure 6(a), top panel), the p56Lck band appeared more intense than the p59Lck band in cells with PHA-P without hTip-CSKH. After 15 min of T-cell stimulation, a progressive increase in p59Lck band staining and tyrosine phosphorylation was noted and this lasted for the rest of the period tested (Figure 6, top left panel). This occurred simultaneously with reduced p56Lck band intensity, suggesting that a more intense p59Lck band depended on the contribution of p56Lck band during PHA-P stimulation. On the contrary, in hTip-CSKH-treated cells, the ratio between p59Lck and p56Lck bands became inverted immediately after stimulation and lasted for the period tested (Figure 6). This rapid increase in Lck phosphorylation after hTip-CSKH and PHA-P stimulation is consistent with the observed increase in proliferation induced by hTip-CSKH in T-cells that have previously been activated with PHA-P (Figure 2). As it would be expected, hTip-CSKH induced on its own Lck phosphorylation and increased tyrosine phosphorylation (Figure 6(c)) in full agreement with the proliferation experiments in Figure 1.

**Figure 7:**
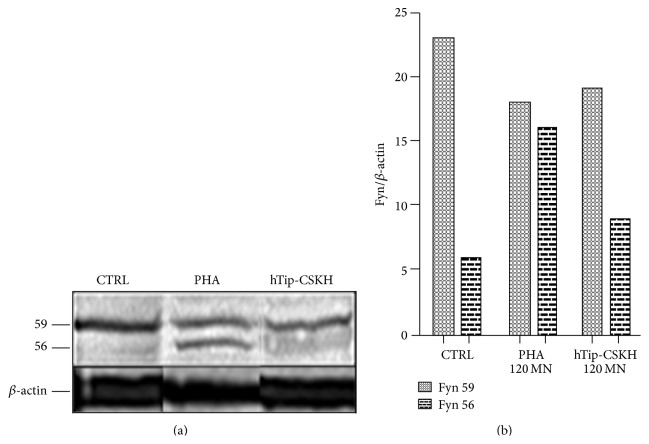
Fyn detection in stimulated human PBMC. (a) PBMC were treated with PHA-P and hTip-CSKH during 2 h, lysed, and the protein extract resolved by SDS-PAGE. Western blot was performed with an anti-Fyn specific antibody. PHA-P was used as positive control, which induces the appearance of a second band of 56 kDa. (b) Quantification of the Fyn p59 and p56 bands. The intensity of each Fyn band was normalized to the loading control *β*-actin.

As it has been reported that Fyn is structurally related to Lck and has at least in part a redundant role during T-cell activation [47], we tested hTip-CSKH effect on this protein tyrosine kinase. Since Fyn activation depends on Lck activation [48] and the latter seems to be still maximal after 1 h of PHA-P or hTip-CSKH stimulation (Figure 6), we tested hTip-CSKH effect on Fyn after 2 h of peptide treatment. As it can be seen in Figure 7(a), PHA-P stimulation induces both the reduction in the intensity of the 59 kDa Fyn protein (by about 22%) and the appearance of a low MW band (56 kDa). hTip-CSKH had a similar effect (20% reduction) on the 59 kDa Fyn protein while the second band appeared diffused and poorly resolved. Thus, hTip-CSKH induces a different effect on Fyn when compared to PHA-P stimulation, suggesting a specificity towards Lck. Additionally, it can be concluded that the increase seen in the Lck band of 59 kDa (Figure 6) is not due to an increase in the Fyn band since, on the contrary, hTip-CSKH induces a reduction in the Fyn 59 kDa band. It might also be considered that although hTip-CSKH induced early activation signals and later proliferation, the signal induced must, of course, be different from the PHA-P signal and far from being complete.

### 3.6. Downstream Signaling Events Induced by hTip-CSKH

To further evaluate hTip-CSKH induced signal strength, we studied Erk phosphorylation, an important step in TCR-induced proliferation [49]. The ERK phosphorylation level achieved during human PBMC stimulation with both PHA-P and PMA was 5.5-fold when compared to control unstimulated cells (Supplementary Figure 3). hTip-CSKH produced 2.5-fold induction in ERK2 phosphorylation; the other peptides tested had minimal or no effect, as evaluated by densitometry. We observed that hTip peptide treatment induced a 1.5-fold increase in ERK1/2 phosphorylation but we considered this effect to be not relevant as it was not enough to induce T-cell proliferation as it was shown above. We conclude then that hTip-CSKH was able to activate signals that are known to occur downstream of Lck.

### 3.7. hTip-CSKH Does Not Downregulate Cell Surface Markers

HVS infection induced a reduction in TCR, CD4, CD3, and CD2 expression. It was shown that this downregulation was partially dependent on Tip interactions with the cellular protein Tap (Tip-associated protein) and mapped to an amino-terminal portion of Tip, different from the Lck binding domain [50]. Therefore, 24-hour hTip-CSKH-treated human PBMC were evaluated for surface marker expression. We observed that CD3 and TCR expression was identical in hTip-CSKH-treated and untreated cells (Supplementary Figure 4), suggesting that the effect of Tip on cell surface receptor downregulation and proliferation could be dissociated by using hTip-CSKH.

## 4. Discussion

HVS infection transforms both non-human primates and human T-cells to TCR-independent proliferation [18–20]. Tip binding to Lck and subsequent Lck activation are partially responsible for this effect [24, 32]. We have previously performed the characterization of Lck in* Aotus nancymaae* and compared them to human Lck. The high identity (98%) found when comparing both species suggests that Lck functionality in* Aotus* T-cells is very similar to humans and that synthetic peptides designed for human Lck binding and modulation could also do so in* Aotus *Lck.

The HVS Tip carboxyl-terminal hydrophobic (hTip) sequence was selected in this study as a cargo vehicle since it has been demonstrated that Tip is constitutively present in lipid rafts [23, 35] and there is experimental evidence connecting hTip with Tip localization in lipid rafts [40]. Additionally, Tip binds specifically to Lck through SH3B and CSKH motifs. Given that a Tip mutant without the SH3B domain was able to transform T-cells [21], we focused on the other Tip motif (CSKH) responsible for Lck binding and activity. It has previously been shown that both motifs can bind Lck independently [32]. Thus, a chimeric peptide formed by hTip and Tip's CSKH motif was synthesized to study its ability to modify T-cell physiology by targeting Lck.

Our results showed a clear increase (2-3 times) in thymidine incorporation in human and* Aotus *PBMC treated with the chimeric hTip-CSKH peptide for 48 h. When longer stimulation periods were used (e.g., 72 h), a 5- to 6-fold increase in proliferation was observed. Taking into consideration that PHA-P induces multiple receptors engagement (with a strong intracellular signaling input), the fact that hTip-CSKH by its own can increase proliferation is remarkable since hypothetically only one specific pathway is being targeted by the use of a unique Lck binding motif (CSKH). Interestingly, after T-cell PHA-P stimulation, hTip-CSKH could further increase proliferation (11–22%), suggesting either a strong hTip-CSKH effect or additional hTip-CSKH stimulation through other mechanisms. This effect was cargo and sequence specific, since control peptides (hTip, hTip-CSKHsc, or CSKH) did not have any effect on proliferation.

Human T-cells require the simultaneous induction of at least two different signaling pathways for full activation, one through the TCR (signal 1) and the second through costimulatory molecules (signal 2). These signals can be mimicked by mitogenic stimulation (PHA-P) or the use of calcium ionophores (signal 1) plus PK-C activation (signal 2) [51]. hTip-CSKH-treated or hTip-treated cells were stimulated with the PK-C activator PMA to test whether signal 2 could further improve hTip-CSKH effect. A 5-fold increase in proliferation was observed with hTip-CSKH, suggesting either that the chimeric peptide is strong enough to induce both signals, as with PHA-P stimulation, or that, under very strong and atypical signal 1 stimulation, signal 2 is not anymore required.

Further evidence of this assumption was obtained by studying hTip-CSKH effect on the T-cell leukemia cell line Jurkat. These cells do not proliferate in response to TCR activation by mitogens (PHA-P) or to anti-CD3 antibodies; even an opposite effect, driving cells to an apoptotic state, has been shown [45]. Tip expression in these cells has also been used as a model for Lck modulation [52]. We have also shown here that hTip-CSKH induces a slight decrease in proliferation, equivalent to PHA-P treatment. This inhibition was more evident and statistically significant when ionomycin-stimulated (signal 1) Jurkat cells were used. It is possible that an apoptosis process is taking place, as in the case of PHA-stimulated Jurkat cells [45]. Thus, if stimulation via the TCR signaling pathway causes cells to proliferate, then hTip-CSKH treatment has a proliferation inducing effect too. On the contrary, if the TCR signaling pathway induces an inhibitory proliferative effect, as what happens in Jurkat cells, hTip-CSKH will also cause this outcome. Although very similar responses to PHA-P stimulation were observed in primary (stimulation) or transformed T-cells (inhibitory) with hTip-CSKH, clearly, an atypical T-cell stimulation by hTip-CSKH will hardly match completely with a classical activation model.

After PBMC hTip-CSKH stimulation and DRM extraction, we could identify the chimeric peptide in fractions containing “heavy” lipid rafts having high content of LAT, Lck, and Ras signaling molecules [46]. Recently, it was shown that an amphipathic helical sequence of 14 amino acids, enriched in charged and hydrophobic amino acids and contiguous to the Tip TM sequence, could increase Tip localization to lipid rafts [40]. Our cargo CSKH sequence has about the same length of this sequence (16 aa) and is also enriched in charged and hydrophobic amino acids, suggesting a similar effect in lipid rafts localization. This membrane microdomain localization was essential to support our initial considerations regarding the use of this hTip sequence and its capacity to deliver a cargo sequence with the capacity to modulate Lck. In fact, we have shown here that hTip-CSKH induces a rapid increase in the appearance of the p59Lck band with a concomitant reduction in p56Lck. These changes are due to Lck phosphorylation and have been previously observed after TCR stimulation [53–55]. This effect was similar in PBMC stimulated only with PHA-P, although the activation kinetics in the presence of the chimeric peptide was extremely rapid. This suggests that hTip-CSKH induces a strong and rapid Lck activation and is responsible on its own for the induced proliferation observed. This is in agreement with the strong proliferation effect of hTip-CSKH shown above.

As Fyn can replace Lck, under certain circumstances, and there is a close sequence and structural identity between both tyrosine kinases, it was interesting to study hTip-CSKH effect on Fyn. Here, a reduction in the p59Fyn band was determined excluding its participation in the observed effect on Lck phosphorylation. The other changes observed in Fyn, especially the appearance of a diffuse p56Fyn band, deserve further experimentation. Nevertheless, these results are in agreement with those that have shown that Tip effect is specific for Lck, not for Fyn or Lyn [25], and consistent with their described differences in protein association and subcompartmental localization [48]. Interestingly, in the TCR signaling models, Fyn activation is dependent on Lck and this process is coordinated spatially in lipid rafts. How this could affect Fyn activation is not known. Since both kinases, Lck and Fyn, have been shown to play a role in the different models of T-cell anergy [56], it would be interesting to explore the hTip-CSKH capacity to revert this process in T-cell.

One of the protein kinases responsible for Lck phosphorylation in Ser residues is ERK, which was activated here too after hTip-CSKH stimulation. As shown before, Lck phosphorylation on Y394 and Y505 is crucial for modulating its activity [57]. Some authors have shown that, after activation, Lck autophosphorylates its Y394 residue further increasing its catalytic activity [58]. We therefore assessed Lck tyrosine phosphorylation and found that p59Lck was more phosphorylated than p56Lck. Taken together, our results have shown that hTip-CSKH induces enhanced T-cell proliferation by targeting and activating Lck. More biochemical and structural work in this field is needed to completely elucidate the mechanism of Lck activation by hTip-CSKH.

By using the hTip-CSKH peptide, the above-described effects on Lck and lymphocyte proliferation could be molecularly separated from the effects caused by other Tip domains. It has been shown that Tip amino-terminal region is particularly involved in cell surface molecules downregulation through Tip binding to a cytosolic p80 protein [23]. We have shown here that surface expression of CD3 and TCR*αβ* was not altered after hTip-CSKH treatment. It was therefore possible to separate the effects induced by Tip CSKH motif from those obtained when the whole Tip molecule was used.

Targeting proteins involved in signal transduction, like protein tyrosine kinases [11, 59] or protein tyrosine phosphatases [60, 61], is considered to be a viable strategy for therapeutic intervention. The use of defined motifs for blocking or inducing a particular function in these signaling molecules is a valuable approach for the fine modulation of cellular processes. We have shown here that the information contained in discrete protein sequences (i.e., CSKH) could be sufficient to modulate complex biological responses, provided that they are duly delivered to specific subcellular compartments. To our knowledge, this is the first report that uses a chimeric peptide to modulate T-cell signaling in lipid rafts. This would undoubtedly be a valuable tool for therapeutic intervention to target molecules whose dynamics depends on lipid rafts.

## 5. Conclusions

We have developed a novel chimeric peptide to modulate Lck signaling in cell membrane lipid rafts. In fact, hTip-CSKH induced on its own strong cell proliferation in normal lymphocytes as a consequence of the specific activation of the lipid rafts-anchored Lck. Interestingly, hTip-CSKH was inhibitory to Jurkat cells, in total agreement with the different signaling pathways and activation requirements of this leukemic cell line. We propose that hTip could be an effective vehicle for delivering a cargo sequence to lipid rafts, and its use could be extended for other molecules responsible for cancer cell growth.

## Supplementary Material

Supplementary Figure 1. Aotus nancymaae (Aona), human (Hosa) and *Saimiri sciureus* (Sasc) Lck alignment of predicted aminoacid sequences. SH3, SH2 and tyrosine kinase domains (boxed and grey) are shown; major phosphorylation sites are labelled with a bold P. Black line in the aminoterminal region shows the conserved sequence used to produce the rabbit antiserum against Lck with synthetic polymeric peptides. Black arrow-heads (12) show the aminoacid position changes between human and *Aotus nancymaae*.
Supplementary Figure 2. Immunofluorescence of human PBMC incubated with FITC-labelled peptides. Cells were incubated with the chimeric hTip-CSKH-FITC peptide and a scrambled CSKH cargo sequence without the hTip domain at 40 μM during 1 h. Samples were analyzed in a Nikon C-1 plus fluorescence microscope and photographed with a Sony DSC-P73 digital camera. Phase contrast and imunofluorescence images are shown.
Supplementary Figure 3. Erk phosphorylation in stimulated human PBMC. A. PBMC were stimulated with the indicated peptides or treatments for 2 h. hTip-CSKH induced an increase (2,5 fold) in ERK2 phosphorylation in human PMBC as shown by WB with anti-pERK1/2 antibody; the membrane was re-probed with anti ERK1/2 for band normalization in each treatment as shown in the bottom graph. DMSO: Dimethyl sulfoxide. PHA+PMA: phytohemaggluttinin + phorbol myristate acetate.
Supplementary Figure 4. CD3 and TCR cell surface determination. Flow cytometry of human PBMC incubated for 24 h in the absence or presence of hTip-CSKH (60 μM). Cells were stained for CD3 and TCR αβ with specific monoclonal antibodies.
