# Supplementary material for: Modulating p56Lck in T-Cells by a Chimeric Peptide Comprising Two Functionally Different Motifs of Tip from Herpesvirus saimiri
Source: J Immunol Res. 2015 Oct 11;2015:395371. doi: 10.1155/2015/395371 (PMC4619936; doi:10.1155/2015/395371)

Supplementary Figure 1

```
                                                  P                 P
                                         ▼   ▼            ▼                         ▼              ▼       ▼  ▼
Hosa MGCGCSSHPE DDWMENIDVC ENCHYPIVPL DGKGTLLIRN GSEVRDPLVT YEGSNPPASP LQDNLVIALH SYEPSHDGDL GFEKGEPLRI LEQSGEWWKA  [100]
Aona .......... .......... .......... ...A...F.. .........R .......... .......... ..K....... ......Q... ...N..R...  [100]
Sasc ........L. .......... .......... ...A...F.. .........R .......... .......... .......... ......H... ...N......  [100]

                                                                   P                                     P P
Hosa QSLTTGQEGF IPFNFVAKAN SLEPEPWFFK NLSRKDAERQ LLAPGNTHGS FLIRESESTA GSFSLSVRDF DQNQGEVVKH YKIRNLDNGG FYISPRITFP  [200]
Aona .......... .......... .......... .......... .......... .......... .......... .......... .......... ..........  [200]
Sasc .......... V......... .......... .......... .......... .......... .......... .......... .......... .........S  [200]

                                            ▼▼                              ▼
Hosa GLHELVRHYT NASDGLCTRL SRPCQTQKPQ KPWWEDEWEV PRETLKLVER LGAGQFGEVW MGYYNGHTKV AVKSLKQGSM SPDAFLAEAN LMKQLQHQRL  [300]
Aona .......... .......... .......... ......LS.. .......... .......... .....D.... .......... .......... ..........  [300]
Sasc .......... .......... .......... .......... .......... .......... .....E.... .......... .......... .......K..  [300]

                                                                                                       P
Hosa VRLYAVVTQE PIYIITEYME NGSLVDFLKT PSGIKLTINK LLDMAAQIAE GMAFIEERNY IHRDLRAANI LVSDTLSCKI ADFGLARLIE DNEYTAREGA  [400]
Aona .......... .......... .......... .......... .......... .......... .......... .......... .......... ..........  [400]
Sasc ........E. .......... .......... .......... ........V. ....L..... .......... .......... .......... ..........  [400]

                                                                                  ▼  ▼
Hosa KFPIKWTAPE AINYGTFTIK SDVWSFGILL TEIVTHGRIP YPGMTNPEVI QNLERGYRMV RPDNCPEELY QLMRLCWKER PEDRPTFDYL RSVLEDFFTA  [500]
Aona .......... .......... .......... .......... .......... .......... .......... H..M...... .......... ..........  [500]
Sasc .......... .......... .........M .......... .......... .........P .......... K..MQ..R.. .D........ ..........  [500]

         P
Hosa TEGQYQPQP [509]
Aona ......... [509]
Sasc ......... [509]
```

Supplementary Figure 2

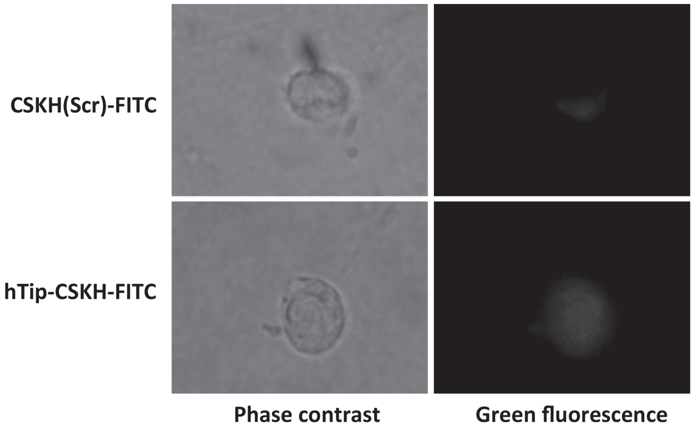

Supplementary Figure 3

A

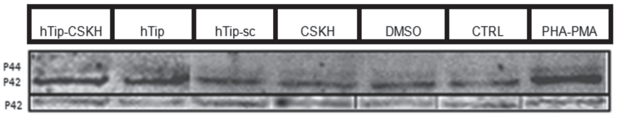

B

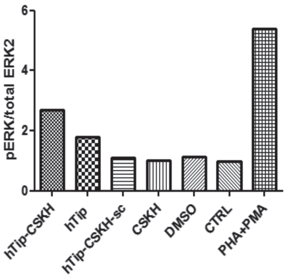

Supplementary Figure 4

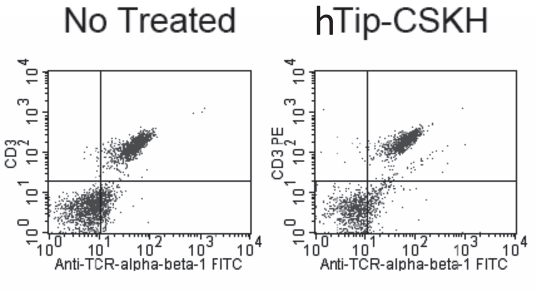

Supplement: Supplementary file 1 — Supplementary Figure 1. Aotus nancymaae (Aona), human (Hosa) and Saimiri sciureus (Sasc) Lck alignment of predicted aminoacid sequences. SH3, SH2 and tyrosine kinase domains (boxed and grey) are shown; major phosphorylation sites are labelled with a bold P. Black line in the aminoterminal region shows the conserved sequence used to produce the rabbit antiserum against Lck with synthetic polymeric peptides. Black arrow-heads (12) show the aminoacid position changes between human and Aotus nancymaae. Supplementary Figure 2. Immunofluorescence of human PBMC incubated with FITC-labelled peptides. Cells were incubated with the chimeric hTip-CSKH-FITC peptide and a scrambled CSKH cargo sequence without the hTip domain at 40 μM during 1 h. Samples were analyzed in a Nikon C-1 plus fluorescence microscope and photographed with a Sony DSC-P73 digital camera. Phase contrast and imunofluorescence images are shown. Supplementary Figure 3. Erk phosphorylation in stimulated human PBMC. A. PBMC were stimulated with the indicated peptides or treatments for 2 h. hTip-CSKH induced an increase (2,5 fold) in ERK2 phosphorylation in human PMBC as shown by WB with anti-pERK1/2 antibody; the membrane was re-probed with anti ERK1/2 for band normalization in each treatment as shown in the bottom graph. DMSO: Dimethyl sulfoxide. PHA+PMA: phytohemaggluttinin + phorbol myristate acetate. Supplementary Figure 4. CD3 and TCR cell surface determination. Flow cytometry of human PBMC incubated for 24 h in the absence or presence of hTip-CSKH (60 μM). Cells were stained for CD3 and TCR αβ with specific monoclonal antibodies. [file 395371.f1.pdf]
